# Characteristics and driving factors of phytoplankton community in urban-rural interface watershed

**DOI:** 10.1038/s41598-026-45995-z

**Published:** 2026-04-02

**Authors:** Lu Wang, Chengjun Wang, Xiaolin Liu, Yuebo Wei, Chunhua Hu

**Affiliations:** 1 Key Lab of Poyang Lake Environment and Resource Utilization, Ministry of Education, Nanchang University, Nanchang, 330029 P. R. China; 2 PowerChina Jiangxi Hydropower Engineering Bureau Co.,LTD, Nanchang, 330096 P. R. China

**Keywords:** Qian Lake water system, Phytoplankton, Community structure, Environmental factors, Redundancy analysis, Dissolved silicon, Ecology, Ecology, Environmental sciences, Hydrology, Limnology, Ocean sciences, Water resources

## Abstract

Urban-rural interface watersheds exhibit distinct ecological characteristics due to the reciprocal influence of human activities and natural processes. The Qian Lake water system, a typical water body at the urban-rural interface, exhibits phytoplankton community characteristics that serve as critical indicators of the region’s ecological health. To examine the community structure of phytoplankton and its environmental drivers in the Qian Lake water system, a systematic survey of phytoplankton and water physicochemical parameters was conducted. Algal biological indicators were used to evaluate the nutrient status of the water body. Redundancy analysis (RDA) was employed to explore the correlations between phytoplankton and environmental factors. The findings revealed a total of 112 phytoplankton species across seven phyla, with Chlorophyta being the most abundant (35.7% of total species), followed by Bacillariophyta, which accounted for 27.7%. Diatoms were the dominant species group (Y = 0.20), likely due to soil erosion caused by suburban expansion and their high efficiency in utilizing dissolved silicon (DSi) in the water. The phytoplankton density ranged from 19.59 × 10^4^ to 61.22 × 10^4^ cells/L, with a mean density of 34.04 × 10^4^ cells/L. The biomass ranged from 0.27 to 0.54 mg/L, with an average of 0.38 mg/L. Furthermore, the community structure of the phytoplankton was of the Bacillariophyta-Chlorophyta type. According to algal biological evaluation criteria, the Qian Lake water system was classified as mildly polluted, and the Pielou uniformity index (J) was less than 0.8, indicating weak stability; RDA and environmental coupling analysis showed that pH, and suspended solids (SS) were the main factors influencing the community structure of phytoplankton in the Qian Lake water system, and the spatial distribution of phytoplankton was co-regulated by nutrient availability, DO availability, and hydraulic conditions (flow velocity, depth of water). These findings provide essential data and theoretical insights for water ecological management and health assessment in urban-rural interface watersheds.

## Introduction

In the complex network of the global ecosystem, phytoplankton, as essential primary producers, play a critical role in the transfer of energy and the circulation of materials^1,2^. Phytoplankton serve as fundamental ecological engines within the vast aquatic domain. Through the process of photosynthesis, phytoplankton absorb and fix carbon dioxide while continuously releasing oxygen. This process not only provides an essential source of oxygen for aquatic organisms but also plays a central role in the global carbon cycle^3,4^.

The survival and development of phytoplankton do not occur in isolation. Instead, their survival and development are comprehensively influenced by a multitude of biotic and abiotic factors^5–8^. Subtle changes in pH can disrupt the acid-base balance both inside and outside phytoplankton cells, thereby affecting their physiological and metabolic processes^9,10^. The intensity and duration of light directly determine the efficiency of photosynthesis in phytoplankton^11,12^. Different species of phytoplankton exhibit varying light requirements and adaptability. For example, Chlorophyta and Bacillariophyta exhibit marked differences in light utilization^13^. Temperature changes influence the accessibility of nutrients and the intensity of light. These changes, in turn, affect the biomass of phytoplankton^14^. For instance, in Lake Wuliangsuhai, the increase in water temperature has positively impacted the density of algae^15^. Macronutrients, particularly nitrogen (N) and phosphorus (P), are critical for the growth and proliferation of phytoplankton^16,17^. When the mass concentration ratio of total nitrogen (TN) to total phosphorus (TP) (TN: TP ratio) is below 7, nitrogen becomes the limiting factor for the growth and development of algae, whereas a TN: TP ratio exceeding 10 indicates phosphorus limitation^18,19^. Håkanson et al.^20^, found that the proportional abundance of Cyanobacteria, compared to that of other algal groups, strongly correlates with the TN: TP ratio. In water bodies with a high TN: TP ratio, Cyanobacteria are more likely to become the dominant population. This demonstrates that changes in the nutrient ratio can significantly affect the species composition and structural configuration of the phytoplankton community. The structure and characteristics of the phytoplankton community are influenced by various environmental factors and reflect the ecological state of the aquatic environment^21^. The structural characteristics of the phytoplankton community are extensively used in the classification and evaluation of aquatic ecological conditions. Common approaches used in the bio-assessment of water quality, based on the structural traits of the plankton community, include the assessment of plankton diversity indices and standing stock^22^; Li and Yu et al.^23^.

As a representative urban-rural interface watershed-defined by intertwined human activities and natural ecological processes (distinguishing it from purely urban or rural aquatic systems)-the Qian Lake water system, situated in a key area of Nanchang City, borders both urban development zones and natural ecological areas. As evidenced by land use dynamics derived from remote sensing imagery between 2018 and 2023, built-up land in the watershed expanded from 63.29% to 74.40% of the total area, while cultivated land decreased sharply from 8.20 km^2^ to 2.10 km^2^ (accounting for only 4.15% of the total area). Furthermore, the water system retains the natural background of the Qian Lake natural lake, yet is subject to intense anthropogenic disturbances from artificially modified channels (e.g., Huanan Canal, Yongqiang Canal) and effluent from wastewater treatment plants, with evident eutrophication observed in recent years. This combination of rapid urbanization, declining rural land cover, and dual ecological pressures renders the Qian Lake water system an ideal site for investigating phytoplankton community dynamics in urban-rural fringe ecosystems. Accordingly, this study focuses on the Qian Lake water system, with the following objectives: (1) to assess the water quality status within the system, (2) to analyze the structural characteristics of the phytoplankton community, and (3) to identify the environmental factors influencing changes in the phytoplankton population structure. The findings of this research contribute valuable references and data for the management of water ecology and the health assessment of urban-rural interface watersheds.

## Materials and methods

### Study area and sampling sites

The Qian Lake water system (115°42′–115°50′E, 28°37′N–28°40′N) is located in Honggutan District, Nanchang. As a key component of Nanchang’s “One River, Three Streams, and Ten Lakes” hydrological network, it has a total catchment area of 61.8 km^2^. The study area is divided into three sections: the upper reach (Huanan Canal: 1.4 km in length; Yongqiang Canal: 3.9 km; Qian Lake Trunk Canal: 2.7 km), middle reach (Qian Lake Reservoir, a natural lake with a total water surface area of 1.66 km^2^, an average water depth of 3 m, and a total storage capacity of 5.8 × 10^6^ m^3^, classified as a small-sized reservoir), and lower reach (Qingyuan Canal: 2.4 km).

The Huanan, Yongqiang, Qingyuan, and Trunk Canals are all artificially modified watercourses. Historically, the surrounding areas were suburban, but with rapid urban expansion, built-up areas have proliferated along the canal banks, particularly in the densely populated upper reach. The Trunk Canal functions as the inflow channel to Qian Lake, receiving water from four sources: the Huanan Canal, Yongqiang Canal, effluent from a wastewater treatment plant, and supplementary water from the Ganjiang River. Hydrological surveys indicate the average flow velocity and daily discharge were 0.027 m·s^− 1^ and 27,081.22 m^3^·d^− 1^ for the Huanan Canal, 0.167 m·s^− 1^ and 16,610.27 m^3^·d^− 1^ for the Yongqiang Canal, respectively. The direct wastewater discharge is 39,044.16 m^3^·d^− 1^, and approximately 1.7 × 10^5^ m^3^ of fresh water is diverted daily from the Ganjiang River. These four sources converge into Qian Lake via the Trunk Canal at a flow ratio of 1:6.13:1.44:6.30.

**Fig. 1:**
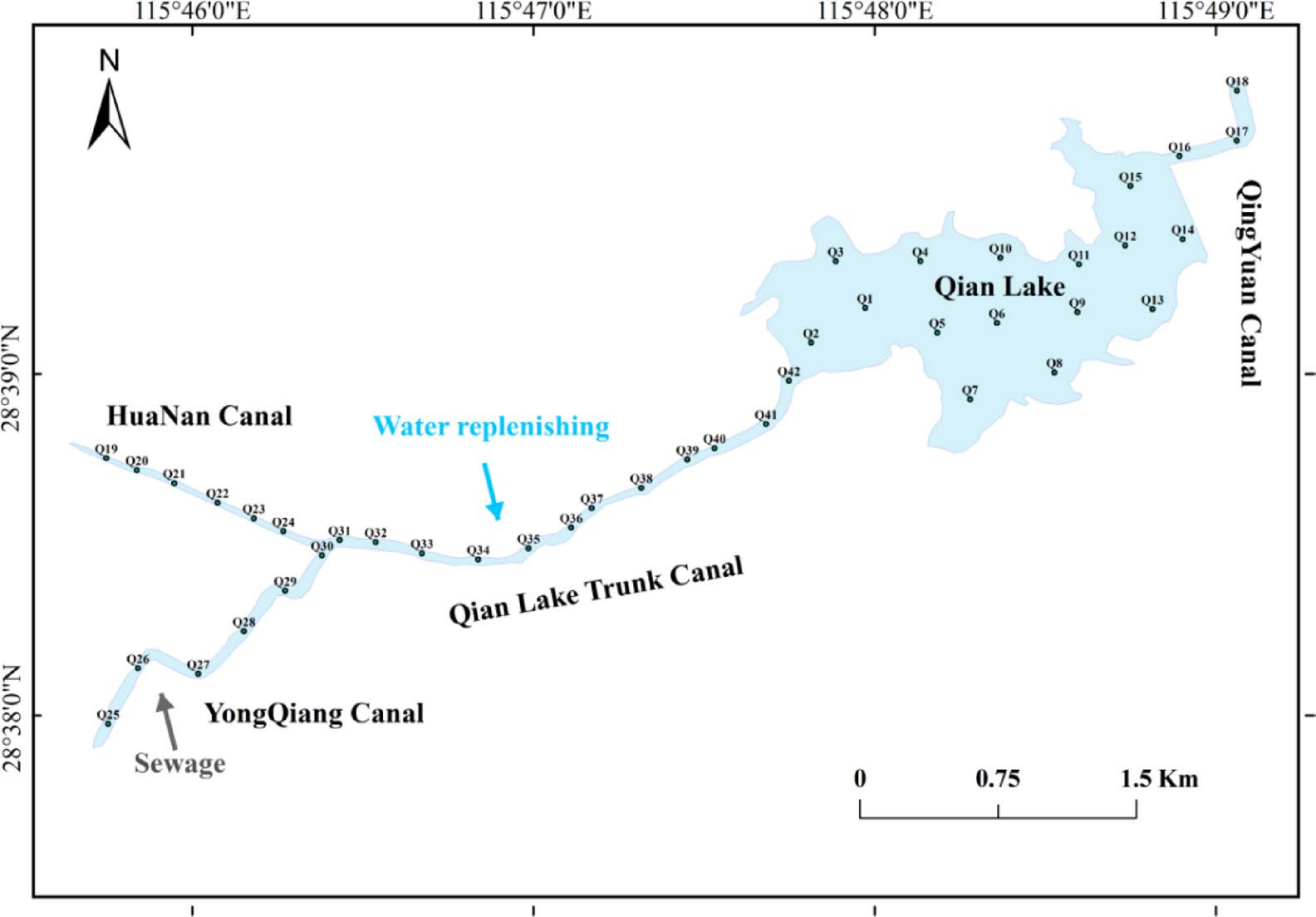
Spatial distribution of sampling points within the Qian Lake water system. (The Huanan Canal and Yongqiang Canal converge and flow into the Qian Lake Trunk Canal, which ultimately leads to Qian Lake. The outlet of the lake is located upstream of the Qingyuan Canal. Upstream of the Yongqiang Canal lies the Jiulonghu Sewage Treatment Plant. The Qian Lake Trunk Canal is supplemented by water diverted from the Ganjiang River).

To investigate the composition of the phytoplankton community and water quality during the stable-water period, 42 sampling sites were established across the Qian Lake water, and water samples were collected on July 10, 2023 (during the stable summer water period of the Qian Lake system, which avoids hydrological fluctuations caused by extreme weather such as heavy rainfall, ensuring the representativeness of phytoplankton and physicochemical parameter data), in accordance with technical guidelines for freshwater biological and lake investigations (Fig. 1). To ensure consistency and clarity in subsequent figures and tables, these sampling sites are systematically abbreviated as HU (Upstream of Huanan Canal), HD (Downstream of Huanan Canal), YU (Upstream of Yongqiang Canal), YD (Downstream of Yongqiang Canal), TU (Upstream of Trunk Canal), TF (Front of the trunk downstream), TM (Middle of the trunk downstream), TB (Backside of the trunk downstream), LU (Upstream of Qian Lake), LM (Middle of Qian Lake), LD (Downstream of Qian Lake), and QY (Qingyuan Canal).

### Sample collection and analysis

Phytoplankton samples were collected for qualitative and quantitative analyses. For qualitative sampling, a #25 plankton net was towed in a figure-eight pattern from a depth of 0.5 m to the water surface (at 20–30 cm/s for 5 min), and captured samples were preserved in qualitative bottles. Quantitative sampling followed the standard method outlined in “Water Quality-Phytoplankton Enumeration by 0.1 mL Counting Frame-Microscope Counting Method” (HJ 1216–2021). Qualitative samples were stored in the dark at 4–10 °C, while quantitative samples were preserved in Lugol’s solution for identification and enumeration. Taxonomic identification was performed according to Freshwater Algae of China: System, Ecology, and Classification^24^. As phytoplankton density was close to that of water, its biomass was calculated using the volume conversion method.

The methods for determining water sample parameters were based on the national standard “Monitoring and Analysis Methods for Water and Wastewater”^25^, which includes pH, dissolved oxygen (DO), suspended solids (SS), electrical conductivity (EC), total phosphorus (TP), total nitrogen (TN), chlorophyll-a (Chl-a), ammonia nitrogen (NH_4_^+^-N) and permanganate index (COD_Mn_). pH, DO, and EC were measured with a YSI multiparameter water quality analyzer.

The Class Ⅳ surface water quality standard (GB 3838 − 2002: Environmental Quality Standards for Surface Water)^26^ was selected as the reference benchmark because it aligns with the designated functional use of the Qian Lake water system (i.e., general industrial water supply and non-direct contact recreational water), a key component of Nanchang’s urban water network. This national statutory standard provides a legally established basis for evaluating whether the water quality meets its ecological and service functions amid rapid urban expansion.

### Phytoplankton community analysis

The dominance index (Y)^27^ was employed to assess species dominance within the biological community, as it comprehensively reflects both numerical dominance and spatial distribution breadth. This index effectively identifies key species that are both abundant and widely distributed. A species was considered dominant when its Y value was ≥ 0.02. The dominance index was calculated according to the following formula:

1 $$\begin{array}{*{20}c} {Y = \frac{{n_{i} }}{N} \times \:f_{i} } \\ \end{array}$$

In this formula, *n*_*i*_ represents the total number of individuals of the i-th phytoplankton species across all sampling sites (Cells); *N* stands for the total number of individuals of all phytoplankton species across all sampling sites (Cells); and *f*_*i*_ denotes the Spatial occurrence frequency of the i-th species, which is the ratio of the number of sampling sites where the species is present to the total number of sampling sites (42 sampling sites in this study).

The Shannon-Wiener diversity index (H’)^28,29^ was employed to assess community diversity, whereas the Pielou evenness index (J)^31^ quantified the distribution uniformity of species within the community. The formulas are as follows:

2 $$H^{{\prime \:}} = - \sum {\:_{{i = 1}}^{S} } P_{i} \log _{2} P_{i}$$

3 $$J = \frac{{H^{\prime}}}{{\log _{2} S}}$$

In this formula, *P*_*i*_ signifies the proportion of species *i*, while *S* represents the total quantity of species. The evaluation criteria are shown in Table 1.

**Table 1:** Evaluation criteria for water quality using plankton diversity indicators^29–32^.

Evaluating indicator	Water quality status				
	High pollution	α-Moderate pollution	β-Moderate pollution	Slight pollution	Clean/no pollution
H′	0 ~ 1.0	1.0 ~ 2.0	2.0 ~ 3.0	3.0 ~ 4.0	≥ 4.0
J	0 ~ 0.3	0.3 ~ 0.5		0.5 ~ 0.8	0.8 ~ 1.0

### Statistical analysis

Statistical analyses were performed using Origin 2021 and ArcGIS for visualization, while Pearson correlation analysis was carried out in SPSS 20.0 to explore the relationships between diversity indices and environmental factors. Redundancy analysis (RDA) was performed using Canoco for Windows 5.0, after confirming its suitability through detrended correspondence analysis (DCA).

## Results and analysis

### Environmental factors of the Qian Lake water system

The key physicochemical and hydrodynamic parameters of the Qian Lake water system are summarized in Table 2. The pH showed only minor fluctuations and remained stable within the range of 7 to 9. DO levels varied widely (1.99–11.08 mg/L), with an average concentration of 6.89 mg/L. The saturation rate notably reached 90% in both the Qian Lake area and the Qingyuan Canal. SS levels ranged from 7 to 9 m, with an average of 8 m. TP levels ranged from 0.10 to 0.69 mg/L (mean: 0.26 mg/L). Yongqiang Canal had the highest TP concentration, peaking at 0.69 mg/L. After water from Huanan Canal flowed into Qian Lake Trunk Canal, TP dropped to 0.42 mg/L (30% lower than Yongqiang Canal’s peak). Following Ganjiang River replenishment, TP in the trunk canal’s lower reaches decreased by 50% versus the upper reaches, and finally fell to the system’s minimum (0.10 mg/L) upon flowing into Qian Lake. In the upstream and middle reaches of the Qian Lake area, as well as the upstream of the Huanan Canal, TP concentrations met the Class IV water quality standard of 0.1 mg/L, while levels in other areas exceeded this threshold. TN concentrations ranged from 1.97 to 10.10 mg/L (mean: 4.67 mg/L), with the mean value exceeding the Class IV threshold of 1.5 mg/L by more than 3-fold and the maximum value by nearly 7-fold. Notably, TN exhibited marked spatial heterogeneity, tributaries like the lower reaches of Huanan and Yongqiang canals had relatively high TN concentrations, with a maximum of 10.1 mg/L. Following the receipt of tributary inflows and Ganjiang River replenishment water, TN concentrations in the Qian Lake Trunk Canal decreased gradually along the flow path, and were significantly reduced in Qian Lake and Qingyuan Canal. Chl-a content ranged from 0.007 to 0.010 mg/L, with an average of 0.008 mg/L. NH_4_^+^-N levels ranged from 0.196 to 7.630 mg/L, with an average of 1.582 mg/L. Concentrations in both the Yongqiang Canal and the upstream of the Trunk Canal exceeded the Class IV water quality standard. The concentration of the permanganate index in the Qianhu water system ranged from 2.3 to 5.2 mg/L, which was lower than 10 mg/L, so the permanganate index in the Qianhu water system reached the standard of lake and reservoir water quality class Ⅳ. The dissolved silicon (DSi) concentration ranged from 2.69 to 5.03 mg/L (mean: 3.80 mg/L). The velocity range of the Qianhu water system was 0.026–0.319 m/s. The depth of water ranged from 0.30 to 3.70 m.

**Table 2:** Physicochemical and hydrodynamic parameters of the Qian Lake water system.

Parameter type		Minimum value	Maximum value	Mean	Class IV of the environmental quality standards for surface Water (GB 3838 − 2002, 2002)
Water quality indicators	pH	7.0	9.0	7.9	6–9
	DO (mg/L)	1.99	11.08	6.89	≥ 3
	SS (m)	7	9	8	–
	EC (*µ*S/cm)	273	447	326	–
	TP (mg/L)	0.10	0.69	0.26	≤ 0.1
	TN (mg/L)	1.97	10.10	4.67	≤ 1.5
	Chl-a (mg/L)	0.007	0.010	0.008	–
	NH_4_^+^-N (mg/L)	0.196	7.630	1.582	≤ 1.5
	COD_Mn_ (mg/L)	2.3	5.2	3.1	≤ 10
	DSi (mg/L)	2.69	5.03	3.80	–
Hydrodynamic parameter	Depth of water (m)	0.30	3.70	2.0	–
	Flow rate (m/s)	0.026	0.319	0.133	–

### Phytoplankton community structure in the Qian Lake water system

#### Phytoplankton species composition and dominant genera

A total of 112 phytoplankton species, representing seven phyla, were identified. Chlorophyta exhibited the highest species richness (40 species, 35.7%), followed by Bacillariophyta (31 species, 27.7%), Cyanobacteria (23 species, 20.5%), Euglenophyta (10 species, 8.9%), Dinophyta (3 species, 2.7%), Cryptophyta (3 species, 2.7%), and Chrysophyta (2 species, 1.8%).

Species with a dominance index ≥ 0.02, along with their corresponding dominance values in the Qian Lake water system, are presented in Table 3. The community structure was characterized by multiple dominant species, with six genera and seven species from three phyla identified as dominant taxa. These taxa included *Oscillatoria tenuis*, *Dolichospermum sp.*, *Raphidiopsis sp.*, *Ulothrix zonata*, *Ulnaria acus*, *Fragilaria capucina*, and *Diatoma sp.*, with dominance indices ranging from 0.02 to 0.06. Cyanobacteria (0.17), Chlorophyta (0.10), and Bacillariophyta (0.20) were the dominant phyla based on species richness, suggesting that the phytoplankton assemblage in the Qian Lake water system during summer was of the Bacillariophyta- Cyanobacteria-Chlorophyta type.

**Table 3:** Dominant species and dominance values of phytoplankton in the Qian Lake water system.

Phylum	Species	Occurrence rate (%)	Y
Cyanobacteria	*Oscillatoria tenuis*	91.67	0.04
	*Dolichospermum sp.*	100.00	0.06
	*Raphidiopsis sp.*	75.00	0.02
Chlorophyta	*Ulothrix zonata*	75.00	0.02
Bacillariophyta	*Ulnaria acus*	83.33	0.05
	*Fragilaria capucina*	83.33	0.04
	*Diatoma sp.*	75.00	0.03

#### Phytoplankton density and biomass

**Fig. 2:**
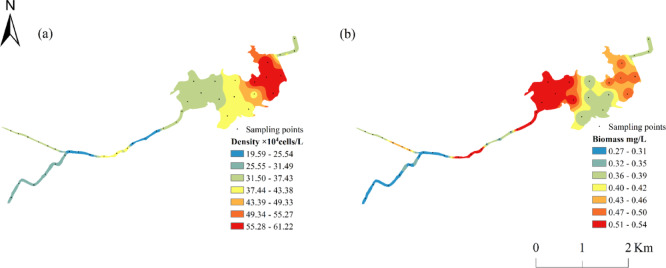
Spatial distribution of phytoplankton density (**a**) and biomass (**b**) in Qian Lake water system.

**Fig. 3:**
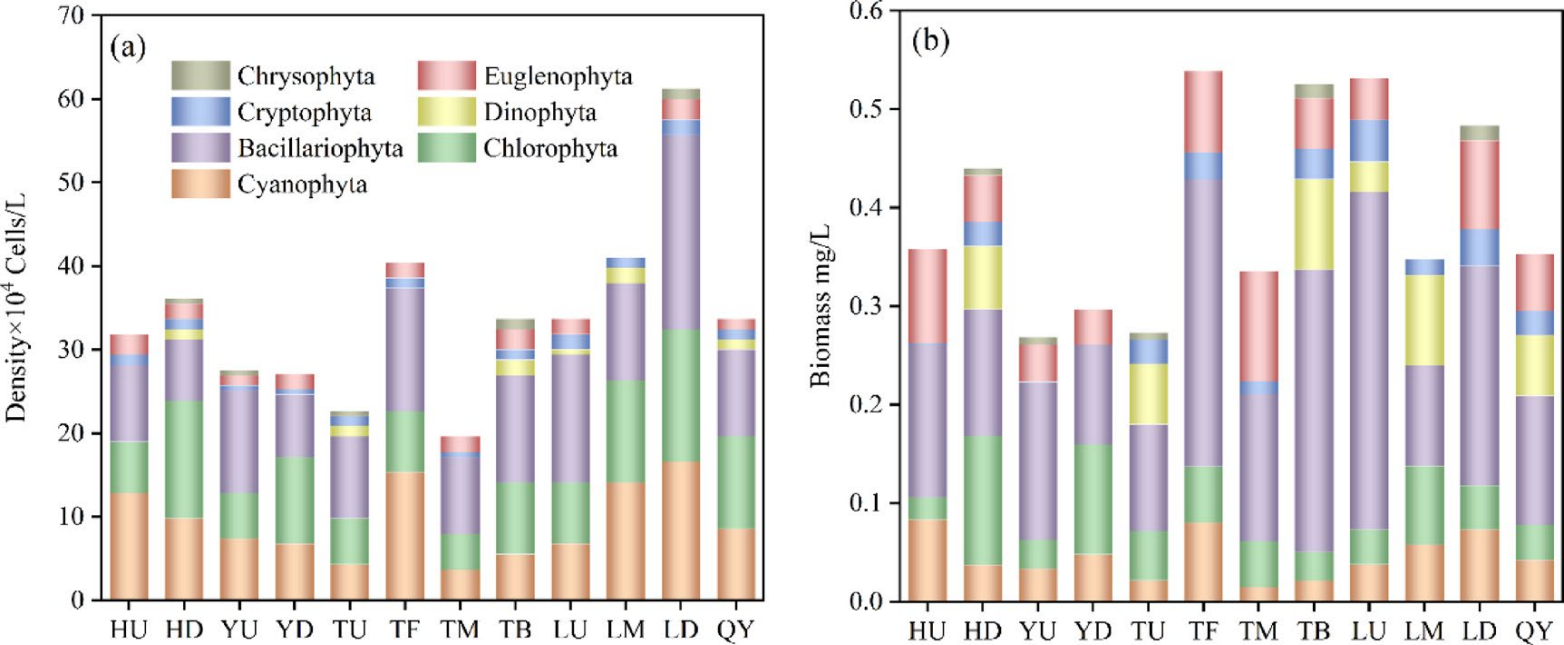
Phytoplankton density (**a**) and biomass (**b**) in the Qian Lake water system.

Phytoplankton density in the Qian Lake water system ranged from 19.59 × 10^4^ to 61.22 × 10^4^ cells/L, with an average density of 34.04 × 10^4^ cells/L, showing spatial variability (Fig. 2a). The highest density was recorded downstream of Qian Lake at 61.22 × 10^4^ cells/L, where the densities of phytoplankton in Cyanobacteria, Chlorophyta, and Bacillariophyta were also the highest among all regions; followed by the lake center at 41.02 × 10^4^ cells/L. The lowest density was observed in the middle reaches of the downstream Trunk Canal at 19.59 × 10^4^ cells/L. Density decreased in the downstream of the Huanan Canal and the Yongqiang Canal but increased after merging into the Trunk Canal of Qian Lake. Following the introduction of water from the Ganjiang River, phytoplankton density gradually increased, peaking in the downstream of Qian Lake (Fig. 3a). Phytoplankton density exhibited an overall gradient of “downstream of Qian Lake> middle of Qian Lake> downstream of the Trunk Canal> upstream of tributaries”.

Phytoplankton biomass ranged from 0.27 to 0.54 mg/L, with an average biomass of 0.38 mg/L, also showing spatial variability (Fig. 2b). The maximum biomass was observed downstream of the Trunk Canal, at 0.54 mg/L, followed by the upstream of Qian Lake with a biomass of 0.53 mg/L. The lowest biomass was observed in the upstream of the Trunk Canal and the Yongqiang Canal (0.27 mg/L for each). Diatoms accounted for the largest proportion of the biomass, followed by Cyanobacteria. Consistent with density, biomass decreased in the downstream of the Huanan Canal and the Yongqiang Canal, then increased after merging into the Trunk Canal of Qian Lake, peaking in the Trunk Canal and Qian Lake (Fig. 3b).

### Relationship between water quality evaluation indicators and environmental factors

**Fig. 4:**
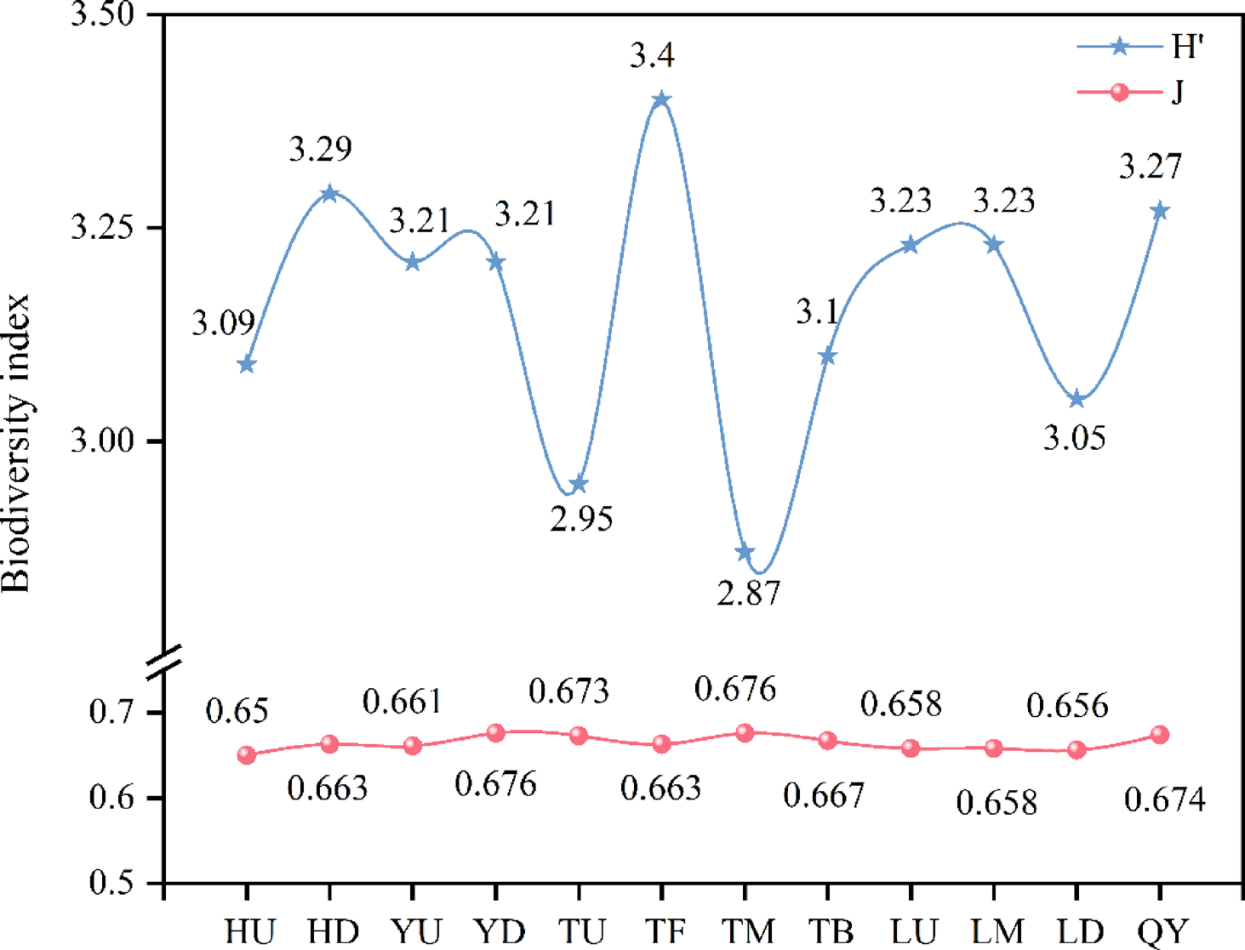
Phytoplankton biodiversity index in the Qian Lake water system.

The higher biodiversity indices generally reflecting better water quality. The trends in phytoplankton diversity and evenness indices within the Qian Lake water system are depicted in Fig. 4. The Shannon diversity index (H’) fluctuated between 2.87 and 3.29, with an average value of 3.15. Only the middle downstream section of the main canal was classified as β-moderately polluted, while the overall water quality was categorized as slightly polluted. The highest evenness index (0.676) was recorded in the middle downstream section of the Trunk Canal, while the lowest (0.650) was observed in the downstream section of the Huanan Canal. No severely polluted areas were identified.

**Fig. 5:**
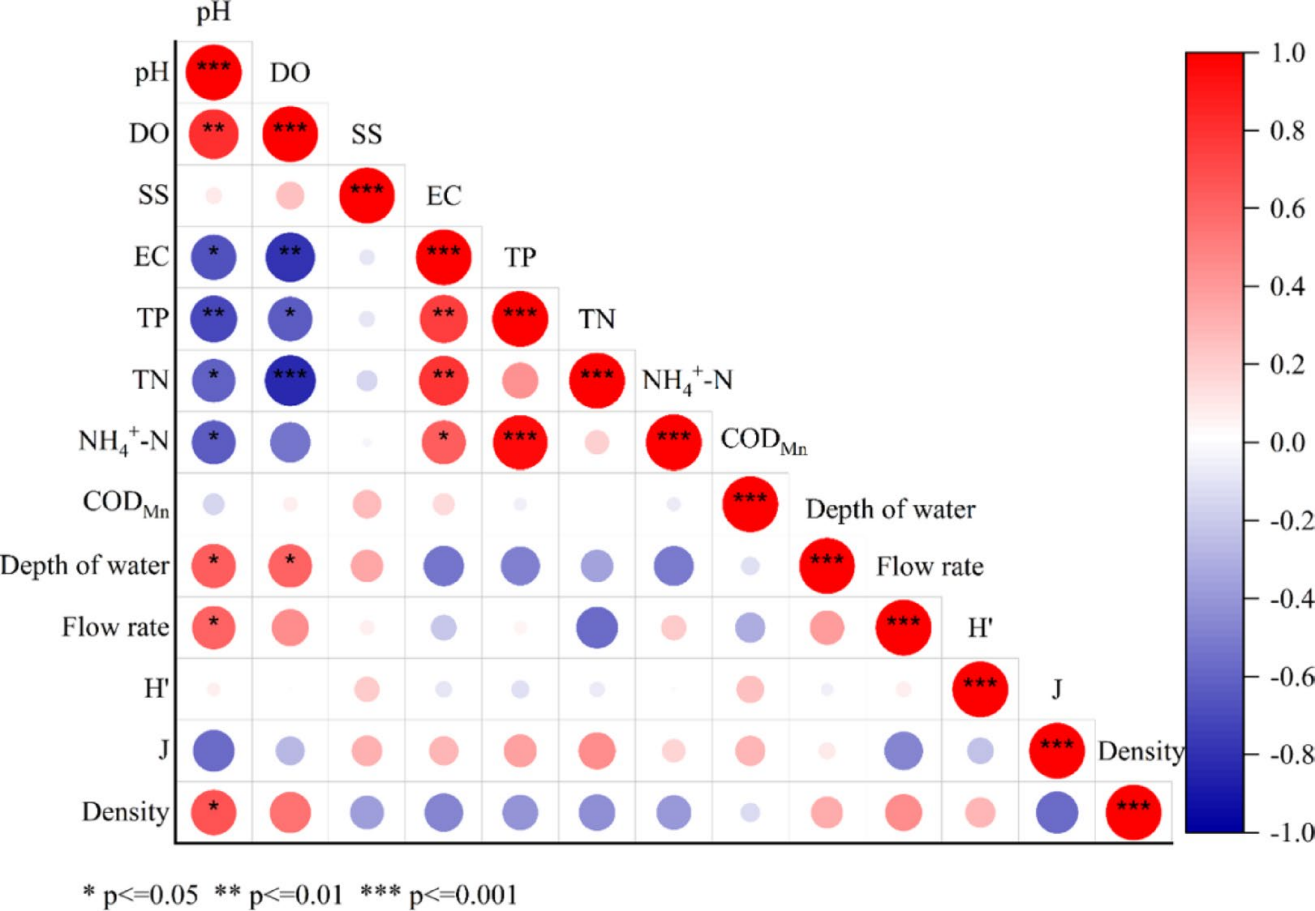
Heatmap of Pearson correlation between species diversity index and environmental factors.

Pearson correlation analysis of phytoplankton density and species diversity indices with environmental factors in the Qian Lake water system is presented in Fig. 5. The diversity index exhibited a weak positive correlation with SS and COD_Mn_. The evenness index showed weak correlations with SS, EC, TN, TP, NH_4_^+^-N and COD_Mn_. Phytoplankton density was significantly correlated with pH (*P* < 0.05) and DO (*P* < 0.1), and weakly correlated with Depth of water and Flow rate. Vigorous photosynthesis of algae during bloom periods increases dissolved oxygen levels, reduces carbon dioxide concentrations, and consequently elevates pH. Additionally, phytoplankton growth was influenced by COD_Mn_, which serves as an indicator of organic pollution.

### Redundancy analysis (RDA) of phytoplankton in relation to environmental factors

Detrended correspondence analysis (DCA) was performed on the cell densities of various algal phyla and ten water quality factors within the Qian Lake water system. The length of the first axis was 0.87 (< 3), indicating that redundancy analysis was appropriate for the dataset. The correlation coefficients of the first and second axes with the abundance of the phytoplankton community were 0.9950 and 0.9974, respectively, suggesting that water quality variables effectively explained the composition of phytoplankton populations in the lake.

**Fig. 6:**
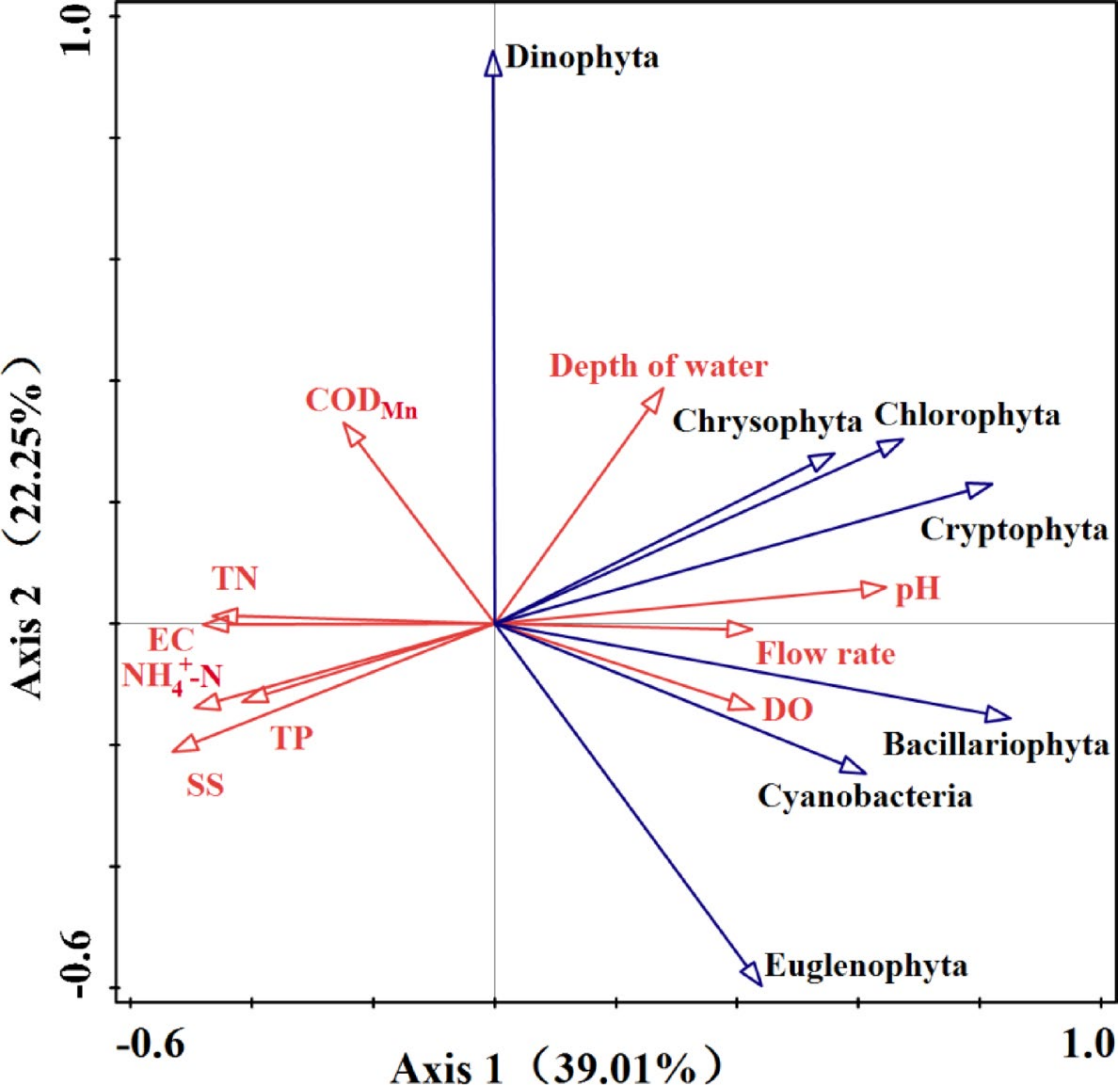
RDA of environmental factors and phytoplankton in the Qian Lake water system.

The correlation coefficients between environmental and species axes were 0.9932 (axis 1) and 0.9965 (axis 2), respectively. The first and second axes had eigenvalues of 0.3901 and 0.2225. These values cumulatively explaining 61.26% of species variation and 67.08% of the species-environment relationship (Table 4). The abundance of the primary algal groups (Cyanobacteria, Chlorophytes, and Diatoms) in the Qian Lake basin was positively correlated with pH, depth of water, flow rate, and DO, and negatively correlated with EC, NH_4_^+^-N, COD_Mn_, SS, TN, and TP. A similar pattern was observed for Chrysophytes, Cryptophytes, and Euglenophytes. In contrast, Dinophytes were positively correlated with pH, TN, EC, COD_Mn_, and Depth of water (Fig. 6).

**Table 4:** RDA results of phytoplankton density and environmental factors.

Statistic	Axis 1	Axis 2	Axis 3	Axis 4
Eigenvalues	0.3901	0.2225	0.1308	0.0982
Explained variation (cumulative)	39.01	61.26	74.34	84.16
Pseudo-canonical correlation	0.9932	0.9965	0.9540	0.9907
Explained fitted variation (cumulative)	42.72	67.08	81.41	92.16

## Discussion

### The characteristics of phytoplankton community structure in urban-rural interface watershed

The phytoplankton community in the Qian Lake water system comprised Bacillariophyta, Cyanobacteria, Chlorophyta, Euglenophyta, Dinophyta, Cryptophyta, and Chrysophyta. The primary components were Bacillariophyta, Chlorophyta, and Cyanobacteria. Chlorophyta accounted for the largest proportion (35.7%), while Bacillariophyta was the most dominant group (Y = 0.20). This compositional characteristic is highly consistent with the dual attributes of “natural ecological processes-human disturbance” in the urban-rural fringe: Chlorophyta species (e.g., Ulothrix zonata) are characterized by high adaptability to nutrient fluctuations and are capable of responding rapidly to short-term nutrient pulses derived from agricultural non-point source inputs in watercourses such as the Yongqiang Canal and Huanan Canal. As a representative of the urban-rural interface watershed, located in northern Jiangxi, the study area’s soil has moderate available silicon (average: 78.4 mg/kg^33^). Meanwhile, urban expansion around Qian Lake led to soil erosion problems. Silicate minerals in the soil released dissolved silicon (DSi) through weathering, which then flowed into the water. Subsequently, DSi was absorbed by Diatoms, promoting their growth^34,35^. As the DSi content decreased from the downstream of the Qian Lake Trunk Canal to Qian Lake, the number of Diatoms increased, indicating that Diatoms could effectively utilize the available DSi^36^. Moreover, Diatoms accounted for a significant proportion in many areas of the Qian Lake water system. For example, the density of Diatoms in the upstream of Qian Lake reached 15.31 × 10^4^ cells/L, far exceeding that of other algae. The high-efficiency ability of Diatoms in utilizing DSi allowed them to gain a competitive advantage over other phytoplankton, which is consistent with findings from the Semerak Lagoon^37^. Dominant species of Cyanobacteria (e.g., Oscillatoria tenuis, *Dolichospermum sp*.) tolerate environments with high NH_4_^+^-N and high COD_Mn_, and adapt to habitats receiving agricultural-domestic mixed pollution inputs, such as the lower reaches of Yongqiang Canal and the upper reaches of the Trunk Canal. In comparison, phytoplankton in Nanhai Lake, a lake with similar characteristics in the urban-rural interface watershed, was predominantly composed of Chlorophyta due to the influence of water pollution and the mesotrophic state of the water body^38^. In contrast, the phytoplankton community in the natural Hongze Lake was dominated by Cryptophyta^39^, while in Chaohu Lake, Cyanobacteria predominated^40^. Zhang^41^, conducted a study on 16 urban lakes and found that Chlorophyta (accounting for 57.19%) was the dominant group in urban lakes. Previous studies have shown that Chrysophytes are dominant in oligotrophic water bodies, Diatoms are more common in mesotrophic water bodies, and Chlorophyta and Cyanobacteria are dominant in eutrophic water bodies^42,43^. This pattern is further supported by empirical evidence from shallow lakes in China, where eutrophication drives the dominance of Cyanobacteria and Chlorophyta, while mesotrophic transitional stages are characterized by Diatom-dominated communities^44^. Based on these findings, it can be inferred that the Qian Lake water system may be in a mesotrophic or eutrophic state. Consequently, the composition of phytoplankton in the urban-rural fringe is consistent with the general characteristics of river and lake systems, being predominantly composed of Diatoms and Chlorophyta^45,46^.

The density and biomass of phytoplankton in the Qian Lake water system exhibited spatial variations across different study areas. Regarding phytoplankton density, the downstream section of Qian Lake had the highest density (61.22 × 10^4^ cells/L), while the middle of the trunk downstream had the lowest density (19.59 × 10^4^ cells/L). This differential distribution indicated that the environmental conditions in different regions significantly influenced the growth and reproduction of phytoplankton. In comparison to the flow velocity of 0.0738 m/s in the middle of the trunk downstream, the downstream area of Qian Lake had a relatively slower water flow velocity of 0.034 m/s, along with a more abundant supply of nutrients (for example, the TN content in the middle of the trunk downstream was twice that in the downstream of Qian Lake), which promoted the aggregation of phytoplankton and facilitated their growth^47^. Phytoplankton density initially decreased in the downstream sections of the Huanan and Yongqiang Canals, then doubled after entering the Qian Lake Trunk Canal, and peaked in downstream of Qian Lake following Ganjiang River water input. This highlighted the critical roles of water system connectivity^48^ and the introduction of water sources^49^ in influencing phytoplankton density. It is likely that the inflowing water brought additional nutrients or altered the hydrochemical and physical characteristics of the water, thereby promoting increased diversity within the aquatic ecosystem and the proliferation of phytoplankton.

Community stability also reflects the ecological vulnerability of urban-rural fringe water bodies. For phytoplankton in the Qian Lake water system, the Shannon-Wiener diversity index and Pielou evenness index were both lower than the thresholds for a “stable community” (H’>3.0, J > 0.8)^50^, with significant spatial variations. Specifically, the lowest evenness (0.663) occurred in the lower reaches of Huanan Canal, corresponding to the local overgrowth of Chlorophyta (accounting for 30% of total phytoplankton in this area); the highest evenness (0.676) was observed in the middle section of the Trunk Canal’s lower reaches, yet actual community stability remained weak due to extremely low density (19.59 × 10^4^ Cells/L). This low stability was closely associated with habitat disturbances in the urban-rural fringe: sediment input (reducing transparency) from urban expansion around Qian Lake, periodic fluctuations in Ganjiang River replenishment (altering flow velocity), and intermittent non-point source pollution (nutrient pulses) from tributaries collectively facilitated local dominance by dominant species, squeezing the niche of sensitive taxa such as Cryptophyta and Chrysophyta^51^. This aligns with the “dominant-species-dominated community” trait of Nanhai Lake (an urban-rural fringe lake) but differs from the stable structure of Hongze Lake (a natural lake)—where Cryptophyta are evenly distributed in summer-further underscoring the uniqueness and vulnerability of phytoplankton communities in urban-rural fringe areas.

### Relationship between phytoplankton community structure and environmental factors in urban-rural interface watershed

#### Coupling relationship between plant plankton community and environmental factors in different regions

**Fig. 7:**
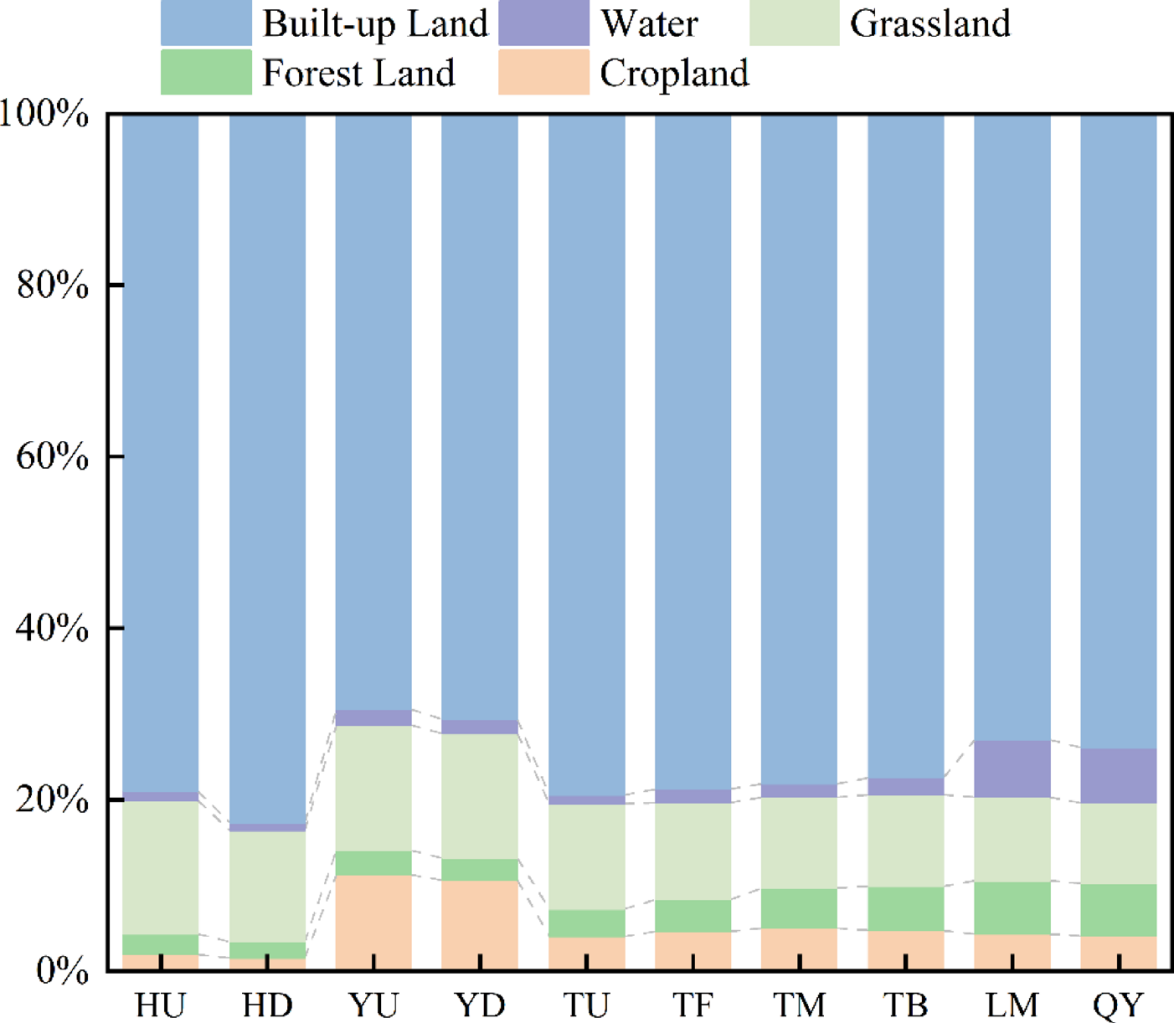
Area ratio of land use types in catchment section of Qian Lake water system.

The RDA results showed that the first two axes explained 61.26% of the total variation in phytoplankton community structure: Axis 1 (39.01%) was primarily driven by nutrient factors (TP, TN, NH₄⁺-N) and SS, while Axis 2 (22.25%) was shaped by hydrological factors (water depth, flow velocity) (Fig. 7). Correlation analysis further confirmed significant associations: phytoplankton density was positively correlated with pH (*P* < 0.05) and DO (*P* < 0.1), but negatively correlated with TP and TN (Fig. 6). Notably, the spatial response of phytoplankton density and biomass to nutrients was not linearly dependent; instead, it was mediated by DO and hydrological conditions, forming a synergistic coupling pattern that varied across hydrological units.

For the Huanan Canal, intense urban land development (built-up land accounting for 79.01% and 82.70% in the upper and lower reaches, respectively) dominates catchment pressure, with minimal agricultural input (cultivated land < 2%) (Fig. 7). This urbanization-driven disturbance shapes local environmental conditions: pH is 8.0, and EC is 350 µS/cm, aligning with the RDA pattern where Chlorophyta and Bacillariophyta (the dominant groups here, with densities up to 14.08 × 10^4^ cells/L and 7.35 × 10^4^ cells/L, respectively) correlate positively with pH and EC (Fig. 6). Low natural vegetation coverage (grassland < 16%) reduces sediment retention, leading to moderate SS that weakly constrains light availability–thus sustaining a relatively stable phytoplankton density (33.97 × 10^4^ cells/L) in the Huanan Canal.

In contrast, the Yongqiang Canal is subject to combined pressures from agricultural non-point sources (cultivated land 11%) and 28% nutrient input from the Jiulong Lake Sewage Treatment Plant. These pressures drive extreme nutrient enrichment, TP reached a maximum of 0.69 mg/L (Upstream of Yongqiang Canal) and TN a maximum of 10.1 mg/L (Downstream of Yongqiang Canal), while DO levels were relatively low (4 mg/L in Upstream of Yongqiang Canal, 4.55 mg/L in Downstream of Yongqiang Canal). As reflected in the RDA (Fig. 6), Cyanobacteria (dominant here, density 6.73 × 10^4^–7.35 × 10^4^cells/L) correlates negatively with TP and TN–this stems from oxygen limitation in high-nutrient environments. Low-DO environments inhibit the electron transfer efficiency of phytoplankton photosystem Ⅱ (PSⅡ) and reduce carbon fixation rates^52–54^. Even with sufficient nutrients, oxygen limitation on phytoplankton proliferation persisted, ultimately keeping phytoplankton density (27.08 × 10^4^–27.55 × 10^4^ Cells/L) and biomass (0.27–0.3 mg/L) in this area at moderate levels. This ecologically validates the negative correlation between phytoplankton and TN/TP observed in the RDA.

In stark contrast to Yongqiang Canal, the lower reaches of the Trunk Canal–having received inflow from Huanan Canal and Ganjiang River replenishment-exhibited significantly reduced nutrient concentrations (TP dropped to 0.21 mg/L, TN to 4.3 mg/L), alongside elevated DO (7.11 mg/L) and slowed flow velocity (0.0738 m/s). Adequate DO provided an aerobic metabolic environment for phytoplankton photosynthesis, while reduced flow minimized hydraulic scouring loss of algal cells, facilitating vertical aggregation and the formation of resource competition advantages. Together, these factors drove phytoplankton density to 40.41 × 10^4^ Cells/L and biomass to 0.54 mg/L, ranking among the highest-biomass zones in the entire water system-highlighting the compensatory effect of “DO-hydraulic conditions” on nutrient limitation.

Qian Lake, the core confluence zone of the water system, exhibited the lowest nutrient levels across the entire system (TP 0.10 mg/L; TN 1.97–2.22 mg/L), alongside high DO concentrations (9.9–11.08 mg/L) and a significantly longer water residence time (29 days) compared to riverine trunk canals. Despite relatively low TN levels, the historical legacy of nitrogen enrichment across the watershed sustains underlying eutrophication pressure, which is amplified by the prolonged water retention time–this extends the window for algae to utilize low-concentration nutrients, driving sustained phytoplankton growth. Elevated DO sustained phytoplankton metabolic activity, while prolonged residence time extended the window for algal uptake of low-concentration nutrients and facilitated population accumulation of dominant taxa such as Bacillariophyta. These combined effects resulted in the highest phytoplankton density in the entire water system (45.30 × 10^4^ Cells/L), reflecting the unique “low-nutrient, high-habitat suitability” ecological advantage of urban-rural fringe lakes. Qingyuan Canal, the receiving waterbody downstream of Qian Lake, had water quality significantly influenced by Qian Lake’s outflow: its nutrient concentrations (TP 0.15 mg/L; TN 2.04 mg/L) and DO level (9.0 mg/L) were all similar to those of Qian Lake, with correspondingly well-matched phytoplankton density (33.67 × 10^4^ Cells/L) and biomass (0.35 mg/L). This further validates the synergistic coupling pattern between nutrients, DO, and phytoplankton spatial distribution.

From a regional comparison perspective, the relationship between phytoplankton communities and environmental factors in urban-rural fringe watersheds (represented by the Qian Lake water system) exhibits both natural attributes and anthropogenic disturbance characteristics. The natural attributes are reflected in the regulation of phytoplankton nutrient utilization efficiency by DO and hydraulic conditions, while the anthropogenic attributes are manifested in nutrient enrichment from agricultural non-point source inputs in Yongqiang Canal, as well as DO elevation and nutrient dilution from Ganjiang River replenishment. This “natural-anthropogenic” dual-driven factor coupling pattern differentiates the local phytoplankton community structure from that of purely natural lakes (e.g., Lake Hongze, where water temperature and TN are the main driving factors)^39^ and urban artificial lakes (e.g., Meixi Lake, where water temperature and redox potential are the main driving factors)^55^, representing one of the typical characteristics of urban-rural fringe aquatic ecosystems.

#### Regulatory role of key factors and eutrophication risk in the Qian Lake water system

The regulatory role of water pH in phytoplankton growth is also critical: pH in the Qian Lake water system remains stable in the weakly alkaline range of 7.0–9.0 and showed the highest explanatory power (20.1%) in RDA analysis. Weakly alkaline conditions promote HCO_3_^−^ dissociation, supplying sufficient carbon for phytoplankton and enhancing algal uptake of nitrogen and phosphorus^56,57^, supporting high phytoplankton density in Qian Lake’s low-nutrient zone. The Chl-a concentration in the Qian Lake water system exhibited a similar variation pattern to phytoplankton biomass, which indicating a correlation between Chl-a levels and phytoplankton growth. This is consistent with the conclusion proposed by García-Nieto et al.^58^, that “Chl-a can serve as a proxy indicator for phytoplankton biomass.” According to the Technical Specification for Eutrophication Assessment of Lakes and Reservoirs formulated by the China Environmental Monitoring Station, a single-index qualitative analysis based on Chl-a (range: 0.007–0.010 mg/L, mean: 0.008 mg/L) indicates the water body is at an oligo-mesotrophic level (≤ 0.010 mg/L).

Nitrogen and phosphorus are essential nutrients required for the growth and propagation of plankton. When the TN content in the water body exceeds 0.2 mg/L and the TP content surpasses 0.02 mg/L, the water body can be classified as eutrophic, with the potential for the occurrence of “water bloom” phenomena^59,60^. Abell et al.^61^, indicated that nitrogen becomes the primary limiting factor for algal growth when the N: P ratio falls below 7. In the Qian Lake water system, average TN and TP concentrations were far above these thresholds, and their N: P ratio greatly exceeded 7. Based on these findings, it is hypothesized that the system has met the conditions for the occurrence of water bloom phenomena, with phosphorus likely serving as the primary limiting factor impeding the growth of phytoplankton. Notably, the extreme nitrogen enrichment is the primary driver of eutrophication potential, even under phosphorus limitation, as sustained nitrogen loading maintains high ecological pressure on the lake ecosystem. Furthermore, phosphorus in freshwater lakes is commonly regarded as a key limiting factor for the development of cyanobacterial blooms^62,63^. The study by Downing et al.^64^, revealed that when phosphorus concentrations are below 0.03 mg/L, the likelihood of Cyanobacteria dominance is relatively low (less than 10%). However, when phosphorus concentrations fall between 0.03 and 0.07 mg/L, the risk of dominance increases to 40%. When the total phosphorus concentration approaches 0.1 mg/L, the risk can exceed 80%. Given that the TP content in the Qian Lake water system generally exceeds 0.1 mg/L, the probability of significant Cyanobacteria proliferation is relatively high, further supporting the dominant role of Cyanobacteria in the Qian Lake water system.

## Conclusions

1. A total of 112 phytoplankton species from 7 phyla were identified in the Qian Lake system. Chlorophyta exhibited the highest species diversity, representing 35.7% of the total species, while Bacillariophyta ranked second with 31 species, accounting for 27.7%. Diatoms, as the dominant functional group (Y = 0.20), likely thrive due to soil erosion from urban expansion and their efficient utilization of dissolved silicon (DSi). Consequently, in the urban-rural interface watershed, Bacillariophyta is the preeminent group among phytoplankton, followed by Chlorophyta, with the community structure predominantly characterized by Bacillariophyta-Chlorophyta.
2. Phytoplankton density in the Qian Lake system ranged from 19.59 × 10^4^ to 61.22 × 10^4^ cells/L, with a mean density of 34.04 × 10^4^ cells/L. Biomass fluctuated between 0.27 and 0.54 mg/L, with an average of 0.38 mg/L. According to algal biological assessment criteria, the Qian Lake system is classified as mildly polluted, with no areas experiencing severe pollution. The uniformity index across the study area was below 0.8, indicating low community stability. This low stability is closely linked to frequent anthropogenic disturbances in the urban-rural interface, including urban expansion-induced soil erosion, non-point source nutrient inputs and water transfer regulation from the Ganjiang River. These disturbances create fluctuating environmental conditions (e.g., variable nutrient loads, altered hydrological regimes) that favor the proliferation of dominant algal species (e.g., Cyanobacteria and Bacillariophyta), thereby reducing community evenness and increasing the risk of algal blooms.
3. The spatial distribution of phytoplankton communities in the Qian Lake water system is jointly determined by nutrients, DO, and hydraulic conditions (flow velocity, depth of water). RDA revealed that pH and SS are the key factors influencing phytoplankton community structure in this system. Specifically, nutrients provide the material basis for phytoplankton growth, while DO and hydraulic conditions regulate algal nutrient utilization efficiency and population aggregation, collectively shaping the community pattern.

## Data Availability

All data generated or analysed during this study are included in this article.The datasets generated and/or analysed during the current study are available from the corresponding author (Dr Chunhua Hu) on reasonable request. Correspondence regarding data access can be directed to: nchuchunhua@163.com (E-mail address of the corresponding author).
